# Immunogenicity of Wild Type and Mutant Hepatitis B Surface Antigen Virus-like Particles (VLPs) in Mice with Pre-Existing Immunity against the Wild Type Vector

**DOI:** 10.3390/v15020313

**Published:** 2023-01-23

**Authors:** Natalie J. Kingston, Renae Walsh, Rachel Hammond, Carina C. D. Joe, George Lovrecz, Stephen Locarnini, Hans J. Netter

**Affiliations:** 1 Infection and Immunity Program, Monash Biomedicine Discovery Institute, Department of Microbiology, Monash University, Clayton, VIC 3800, Australia; 2 School of Molecular and Cellular Biology, Faculty of Biological Sciences, Astbury Centre for Structural Molecular Biology, University of Leeds, Leeds LS2 9JT, UK; 3 Victorian Infectious Diseases Reference Laboratory (VIDRL), Melbourne Health, The Peter Doherty Institute, Melbourne, VIC 3000, Australia; 4 School of Science, Royal Melbourne Institute of Technology (RMIT) University, Melbourne, VIC 3001, Australia; 5 Commonwealth Scientific and Industrial Research Organisation, Clayton, VIC 3169, Australia; 6 Jenner Institute, Nuffield Department of Medicine, University of Oxford, Oxford OX3 7BN, UK

**Keywords:** vectored vaccines, delivery platforms, immune sensitization, hepatitis B surface antigen (HBsAg)

## Abstract

Virus-like particles (VLPs), composed of the small hepatitis B virus surface antigen (HBsAgS), are the antigenic components of the hepatitis B virus (HBV) vaccine and represent the backbones for a chimeric anti-malaria vaccine and various vaccine candidates. Biological vectors have to face pre-existing anti-vector immune responses due to previous immune exposure. Vector recognition after natural infections or vaccinations can result in unwarranted outcomes, with compromising effects on clinical outcomes. In order to evaluate the impact of a pre-existing anti-HBsAgS immune response, we developed mutant VLPs composed of subunits with reduced HBsAgS-specific antigenicity. The insertion of a *Plasmodium falciparum* circumsporozoite protein (CSP)-derived epitope as a read-out allowed the assessment of wild type (wt) and mutant VLPs in the context of a pre-existing immune response. Mutant and wt VLP platforms with a CSP-epitope insert are immunogenic and have the ability to generate anti-CSP antibody responses in both naïve BALB/c mice and mice with a pre-existing anti-HBsAgS immune response, but with superior anti-CSP responses in mice with a pre-existing immunity. The data indicate that previous HBsAgS exposure facilitates enhanced antibody responses against foreign epitopes delivered by the HBsAgS platform, and, in this context, the state of immune sensitization alters the outcome of subsequent vaccinations.

## 1. Introduction

Live attenuated bacterial and viral vectors including non-infectious carrier platforms, have been developed as delivery systems for medically important antigenic sequences [1,2]. Biological vectors and platforms have to face pre-existing anti-vector or anti-platform immune responses, which can result in unwarranted outcomes with compromising effects on clinical outcomes [3]. Pre-existing immunity against the human adenovirus serotype 5 (hAd5) vector was associated with an elevated acquisition of the human immunodeficiency virus (HIV) in vaccinated participants, compared to a placebo [4,5]. A pre-existing yellow fever vaccine-derived immunity generates a negative effect on the antibody response to the tick-borne encephalitis (TBE) virus. The impairment of the neutralizing antibody response to TBE vaccination is possibly due to the antigenic relatedness of flaviviruses [6]. Depending on the selected vector systems, the impact of a pre-existing anti-vector immune response results in opposing immunization outcomes, possibly due to differences in the inherent immunogenicity of the used carrier molecules. This strongly suggests that vector–antigen relationships must be individually assessed [7,8]. Various interference mechanisms have been associated with multivalent glycoconjugate vaccines, and evidence for carrier priming or suppression has been reported [9,10]. Carrier-induced epitope-specific suppression was evident in studies using peptides conjugated to the tetanus toxoid. The immunization of mice with the tetanus toxoid inhibited subsequent antibody responses to the peptide conjugated to the tetanus toxoid carrier [7], which seems to be mediated through clonal dominance facilitated by the expansion of carrier-specific B-cells [11,12]. An increased hapten density prevented the induction of epitope suppression [11,13,14]. To minimize the impact of pre-existing vector immunity in humans, vectors have been developed which are not associated with human infections or from viruses of rare serotypes. Alternative strategies involve heterologous prime-boost approaches, homologous reimmunization, or removing key neutralizing epitopes [3,4,15,16]. 

One of the most successful VLP-based vaccines is the hepatitis B virus (HBV) vaccine [17,18,19]. The HBV vaccine is based on the small hepatitis B surface antigen (HBsAgS) VLPs, which are the sole antigenic components required for the generation of protective immunity against all HBV serotypes. Particle formation requires the cotranslational insertion of HBsAgS into the ER membrane with a short luminal-exposed N-terminal sequence, two transmembrane regions separated by a cytosolic loop, and a luminal external domain containing the major B-cell epitopes (‘a’-determinant). The surface-exposed region is proposed to contain two extracellular loops shaped by disulfide bonds [17,20]. The VLPs are 22–25 nm in diameter, and it is estimated that one particle contains approximately 100 HBsAgS molecules. The compact structure is due to the large number of intra- and intermolecular disulfide bonds within and between the individual subunits [17,21] and imparts ideal properties for the use of VLPs as vaccine platforms. HBsAgS VLPs accept the insertion of foreign antigenic sequences, providing the basis for delivery platforms, as in the case of the RTS,S/AS01 (Mosquirix™) vaccine against malaria [17,22,23]. Mosquirix™ encodes 189 amino acids of the *Plasmodium falciparum* circumsporozoite protein (CSP) fused to the N-terminus of the HBsAgS. Alternatively, to promote the surface orientation of the introduced B cell epitopes, foreign sequences were inserted into the external domain of HBsAgS VLPs and successfully used for the generation of anti-insert-specific immune responses [24,25,26,27,28]. The insertion of a nine-mer “Asn-Ala-Asn-Pro” (NANP)-repeat (NANP9) from the *P. falciparum* CSP protein allowed the induction of anti-CSP antibodies with biological activity [29]. 

The high number of vaccinated individuals, and also the occurrence of acute hepatitis B, leads to a global population with a high prevalence of seropositivity against HBsAg proteins [30]. Therefore, the impact of a pre-existing anti-HBsAgS immune response on the efficiency of HBsAg platforms needs to be understood. The insertion of a foreign sequence into the ‘a’-determinant region reduces the HBsAgS-specific antigenicity [25,29] but retains HBsAgS-specific immunogenicity, indicated by the induction of anti-HBsAgS-specific antibodies [26,29]. To assess the contribution of the HBsAgS scaffold, we have generated chimeric NANP9-containing VLPs composed of subunits with key HBsAgS-specific antigenic sites mutated. Using either wild type (wt) or antigenically mutant VLPs as scaffolds for the *P. falciparum*-specific NANP9 insert for immunization studies in naïve or anti-HBsAgS positive BALB/c mice demonstrated that the wt and mutant VLP platforms are immunogenic. However, the use of the wt HBsAg backbone in the presence of a pre-existing anti-HBsAgS antibody response promoted the anti-NANP9 immune response. The high antigenic density of the inserted NANP9 epitopes and the enhanced recognition of the wt HBsAgS backbone compared with the mutant HBsAgS backbone in the context of pre-immune B-cell repertoire appears to facilitate a superior anti-NANP9 antibody response.

## 2. Materials and Methods

### 2.1. Plasmids

The parental expression plasmid has been previously described and encodes an N-terminally myc-tagged HBsAg protein (genotype D, serotype *ayw*) with a *P. falciparum*-specific epitope (NANP9) inserted into the ‘a’-determinant region (HBsAgS M1-protein) [29]. The HBsAgS cDNA was mutated to generate HBsAgS proteins with reduced HBsAgS-specific antigenicity; M1-HBsAgS represents the wt protein with an N-terminal myc-peptide tag (EQKLISEEDL). M15-HBsAgS proteins contain seven selected mutations and one deletion, as specified in Supplementary Table S1, to minimize the HBsAgS-specific antigenicity. The NEBaseChanger and the Q5 Site-Directed Mutagenesis Kit were used to design and generate the mutant cDNA, as per the manufacturer’s instructions (New England Biolabs, Ipswich, MA, USA). In addition, the M15-HBsAgS was also expressed as an HA-tagged version and accordingly named H15-HBsAgS. 

### 2.2. Immunoanalyses of HBsAgS Proteins

The myc-tagged wt M1- and myc-tagged mutant M15-HBsAgS proteins were expressed in human embryonic kidney HEK293T cells transfected with the appropriate expression plasmids using polyethylenimine (PEI, Polysciences Inc., Warrington, PA, USA), as previously described [29,31]. A total of 3 × 10^5^ cells were seeded into each well of a 6-well tissue culture dish (BD Bioscience, Bedford, MA, USA) and transfected with 3 μg plasmid DNA the following day. After two days, the cell culture medium was harvested and assessed for the presence of myc-tagged HBsAgS proteins by ELISA. The cells were incubated in cysteine- and methionine-free DMEM (Dulbecco’s modified Eagle’s medium) for 45 min before the addition of 200 mCi ^35^S-labelled cysteine and methionine (Perkin Elmer, Waltham, MA, USA). The cells were incubated for 3 h; then, the medium was replaced with DMEM supplemented with 10% fetal calf serum (FCS) and incubated for 18 h. The cell culture medium was harvested and the cells lysed in 200 μL NP40 lysis buffer (150 mM NaCl, 1% NP40, 50 mM Tris, pH8). Iodoacetamide at a final concentration of 20 mM was added to the cell culture medium and diluted lysate, then incubated on ice with the mouse monoclonal anti-myc antibody (9E10, Invitrogen, Waltham, MA, USA) or polyclonal rabbit anti-HBsAgS antibodies (Meridian Life Science, Memphis, TN, USA) in the presence of protein A sepharose. After incubation at 4 °C with rapid agitation, the samples were washed three times with RIPA buffer (150 mM NaCl, 1% NP-40, 1% sodium deoxycholate, 0.1% SDS, 10 mM Tris-HCl pH 7.5), and once with 100 mM Tris-HCl, pH6.8. The samples were denatured in Laemmli buffer, separated by 15% SDS-polyacrylamide electrophoresis (SDS-PAGE), fixed, dried, and exposed on a Typhoon Phosphorimager (GE Healthcare, Chicago, IL, USA). 

The cell culture medium collected before the ^35^S-labelling step was analyzed for the presence of HBsAgS proteins using an HBsAg-specific diagnostic assay (MonoLISA, Bio-Rad, Berkeley, CA, USA) (as per the manufacturer’s instructions) and an ELISA for the detection of the myc-tag. For the anti-myc ELISA, 100 μL of 500 ng/mL rabbit polyclonal anti-myc antibodies (Sigma-Aldrich, St. Louis, MO, USA) were coated onto a 96-well plate (Nunc MaxiSorp, Thermo Scientific, Waltham, MA, USA), incubated overnight at 4 °C, and blocked with skim milk powder in phosphate buffer salt (PBS); then, the sample cell culture medium was added. Myc-tagged HBsAgS proteins were detected with a mouse monoclonal anti-myc antibody (9E10) and rabbit polyclonal anti-mouse IgG antibodies conjugated to horseradish peroxidase (HRP) (Dako, Agilent Technologies, Glostrup, Denmark). The plates were incubated with 2,2′-azino-bis(3-ethylbenzothiazoline-6-sulphonic acid) (ABTS), H_2_O_2_ substrate, and the optical density was measured at 405 nm.

### 2.3. Multiplex Immunoassay

A Bio-Plex bead-based flow cytometric platform (Bio-Rad, Hercules, CA, USA) was established for the characterization of the antigenic profile of the HBsAgS VLPs [31,32]. The assay utilizes a set of different anti-HBsAgS antibodies, each antibody type conjugated to fluorescently tagged beads, and a polyclonal phycoerythrin-conjugated detector antibody. Seventeen monoclonal anti-HBsAgS antibodies were used to test epitope-binding to the mutant HBsAgS VLPs with the following binding specificities, monoclonal antibody (mAb) 1: N-terminus; mAbs 5 and 10: loop 1; mAbs 8, 11, 12, 16, 17 and 19: loop 2; mAbs 13, 14 and 15: loop 1 and loop 2; mAbs 2, 3 and 4: C-terminus; mAbs 9 and 18: conformational. The origin of the mAbs is acknowledged by Hyakumura et al. (2015) [31], and an illustration of the antibody-binding sites was published by Walsh et al. (2019) [32]. The 95% confidence interval (CI) for the normal range of variation of epitope recognition from the reference backbone was established as ±0.5-fold change. A fold change <0.5 was considered insignificant, reflecting the normal variance of this assay. The positive fold changes (>0.5-fold) and negative fold changes (>0.5-fold) corresponded to a gain or reduction of epitope binding, respectively. A threefold reduction was considered a complete epitope knockout.

### 2.4. Large-Scale HBsAgS VLP Production 

The large-scale production of HBsAgS VLPs for the immunization studies was performed as previously reported [29]. Suspension-adapted HEK293F cells were grown in DMEM and 10% FCS in a 1 L wave bioreactor, and transfected with lipofectamine according to the manufacturer’s instructions (Life Technologies, Carlsbad, CA, USA). The tissue culture medium was harvested, centrifuged to remove any cellular debris, and then the HBsAgS VLPs were pelleted by ultracentrifugation in the presence of a 20% sucrose cushion in STE (100 mM NaCl, 1 mM EDTA, 10 mM Tris, pH8), as described [31]. The pelleted VLPs were resuspended in 200–500 μL STE. The VLPs containing the NANP9 inserts were quantified relative to the M1-HBsAgS particles via the myc-tag [29].

### 2.5. Immunization and Assessment of Antibody Responses

BALB/c mice were used at the age of week 6. The mice were housed under pathogen-free conditions at a facility of the Monash Animal Research Platform, and the experiments were approved by the animal ethics committee. The BALB/c mice were immunized subcutaneously at the base of the tail with 2 μg VLPs in the presence of 2.5 nmol CpG (ODN1668), as previously described [29]. The mice were immunized four times in two-week intervals (days 0, 14, 28, and 42) and the last bleed was taken two weeks after the fourth immunization (day 56). For studies in the presence of a pre-existing anti-HBs antibody response, the BALB/c mice were immunized three times with wt, yeast-derived VLPs (days 0, 14, and 28) followed by immunization with 2 μg NANP9-containing VLPs composed of wt M1-HBsAgS or mutant M15-HBsAgS proteins in the presence of 2.5 nmol CpG. The immunization schedule is shown in Supplementary Table S2. 

For the assessment of the anti-HBsAgS immune response, an ELISA was performed using wt, yeast-derived HBsAgS VLPs (Meridian, serotype *ayw*) as targets. The VLPs were coated onto ELISA plates (Nunc MaxiSorp) at a volume of 100 μL (1 μg/mL) overnight at 4 °C, then blocked with 300 μL of 5% skim milk in PBS for 2 h at room temperature, followed by a 1 h incubation with the mouse sera, and diluted in PBS at room temperature. The plates were washed, then incubated with 100 μL (1 μg/mL) anti-mouse IgG coupled to HRP, washed again, and the presence of anti-HBsAgS antibodies visualized in the presence of an ABTS substrate solution, and the optical density recorded at OD 405–490 nm. Alternatively, the presence of anti-HBsAgS was determined using the anti-HBsAg diagnostic testing platform (Elecsys^®^, Anti-HBs, Roche, Rotkreuz, Switzerland) with the Cobas e411 analyzer (Roche), and documented as international units per liter (IU/L). For determining mutant M15-HBsAg-specific antibody activity, the immune sera were tested against HA-tagged mutant H15-HBsAgS VLPs containing the identical mutant HBsAgS sequence as M15-HBsAgS. 

## 3. Results

### 3.1. Generation of Antigenically Distinct HBsAg VLPs

**Figure 1:**
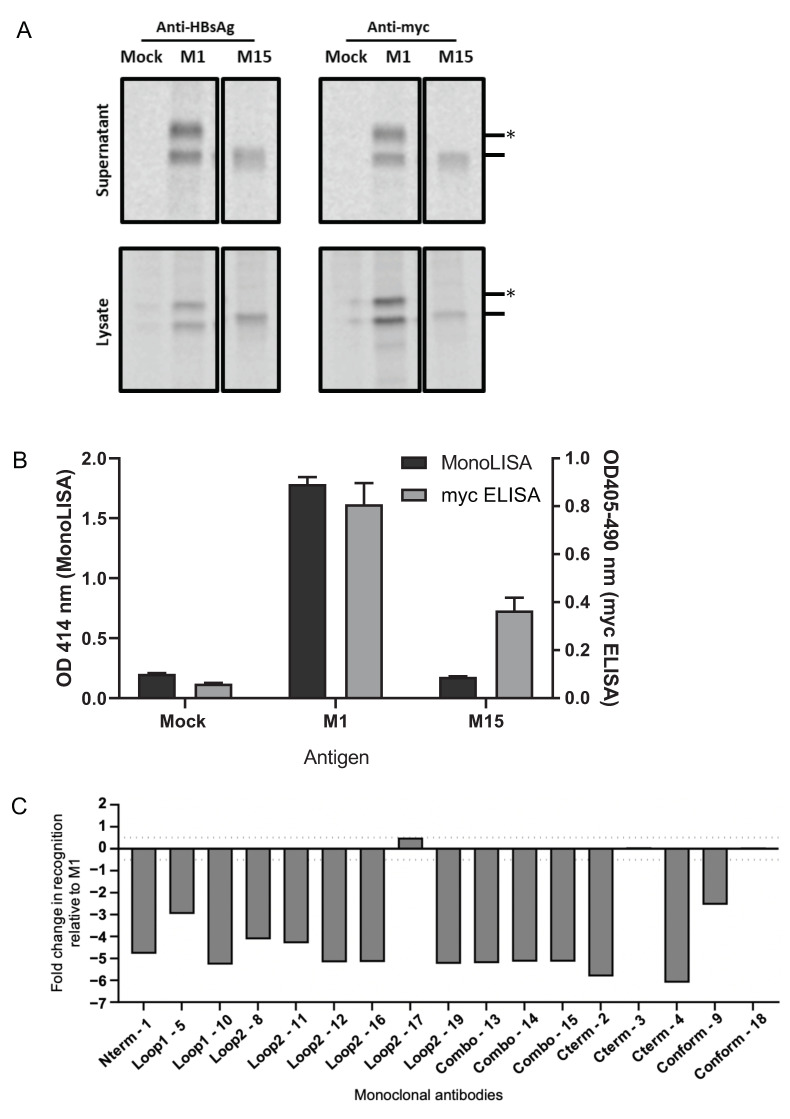
Expression and secretion of myc-tagged M1- and M15-HBsAgS proteins produced in HEK293T cells. Cells were transfected with plasmids expressing M1- or M15-HBsAgS. As a control, cells were mock-transfected. Detection of ^35^S-labelled HBsAgS protein in the cell culture medium and cell lysate by immunoprecipitation with (**A**) polyclonal anti-HBsAg and anti-myc monoclonal antibodies. Protein (dash) and glycoproteins (asterisk) are indicated. (**B**) Anti-HBsAg (MonoLISA, Bio-Rad) and anti-myc ELISA using cell culture medium, *n* = 3, in duplicate, graphed mean and standard error of the mean (SEM). (**C**) The HBsAg-specific antigenicity of M15-HBsAgS was assessed relative to M1-HBsAgS using the Bio-Plex Assay. Antibodies directed against the N-terminal region, loop 1, loop 2, combination of both loops (Combo), C-terminal region, and conformational epitopes (Conform) were used to assess antigenic changes across the length of the modified HBsAg proteins. Antigenic reactivity between 0.5 and −0.5 is considered the normal variation of this assay; values greater or less than this are considered significant antigenic changes; values less than −3 are considered complete epitope losses.

To address the impact of pre-existing anti-HBsAgS antibodies on immunization outcomes, wild type (wt) and mutant HBsAgS VLPs with reduced HBsAgS-specific antigenicity were generated. The HBsAgS-immunodominant region was mutated, and the amino acids were deleted to minimize the HBsAgS-specific antigenicity. The changes include cysteine-to-alanine changes in the loop 1 region to interfere with the correct HBsAgS folding by altering the disulfide bonding, the deletion of the N146 glycosylation site, and the introduction of the G145R mutation, which is associated with vaccine escape [33,34], among other mutations (Table S1). The HBsAgS proteins were N-terminally tagged with the myc-peptide tag for monitoring HBsAgS expression independent of altered antigenicity. The myc-tagged wt proteins (M1-HBsAgS) and mutant proteins (M15-HBsAgS) were expressed in HEK293T cells, ^35^S-labelled, and then immunoprecipitated from the cell culture medium and cell lysate using monoclonal anti-myc or polyclonal anti-HBsAgS antibodies (Figure 1A). As expected, the wt M1-HBsAgS proteins are separated as glycosylated (27 kDa) and non-glycosylated (24 kDa) versions; in contrast, the M15-HBsAgS proteins are not glycosylated, due to the absence of the N146 glycosylation site showing only a protein at 24 kDa (Figure 1A). Unlabeled HBsAgS proteins were collected from the cell culture medium, before the ^35^S-label was added, and assayed for the presence of the HBsAgS protein using a myc-specific ELISA and an established HBsAgS-specific assay (MonoLISA, Bio-Rad). The anti-myc antibodies detected M1 and M15-specific proteins in the cell culture medium; in contrast, the HBsAgS-specific MonoLISA assay identified wt M1-HBsAg proteins, similar to the myc-specific ELISA, but failed to detect the mutant M15 proteins, strongly indicating that the M15 proteins had lost HBsAgS-specific antigenicity in this assay (Figure 1B). The extent of HBsAgS-specific epitope loss was determined by a multiplex assay using 17 anti-HBsAgS monoclonal antibodies and measured as the change in binding relative to the control, the myc-tagged wt HBsAg (M1) backbone (Figure 1C). Fourteen monoclonal antibodies did not detect the M15 VLPs, relative to the myc-tagged wt VLPs, indicating a substantial loss of HBsAgS-specific antigenicity. Only three monoclonal antibodies bound to the M15 proteins, indicating that some HBsAg-specific antigenic sites had been retained. The C-terminal-specific antibody mAb3, and the conformation-dependent antibodies mAb18 and mAb17, which bind to the external loop region, still detected the M15. This is consistent with the detection of M15-HBsAgS proteins by the polyclonal anti-HBsAg serum, which indicates that some HBsAgS-specific epitopes had been retained (Figure 1A), in spite of a substantial loss of HBsAgS-specific antigenicity (Figure 1C).

### 3.2. Determination of the Immunogenicity of Myc-Tagged wt M1 and Myc-Tagged Mutant M15 VLPs

**Figure 2:**
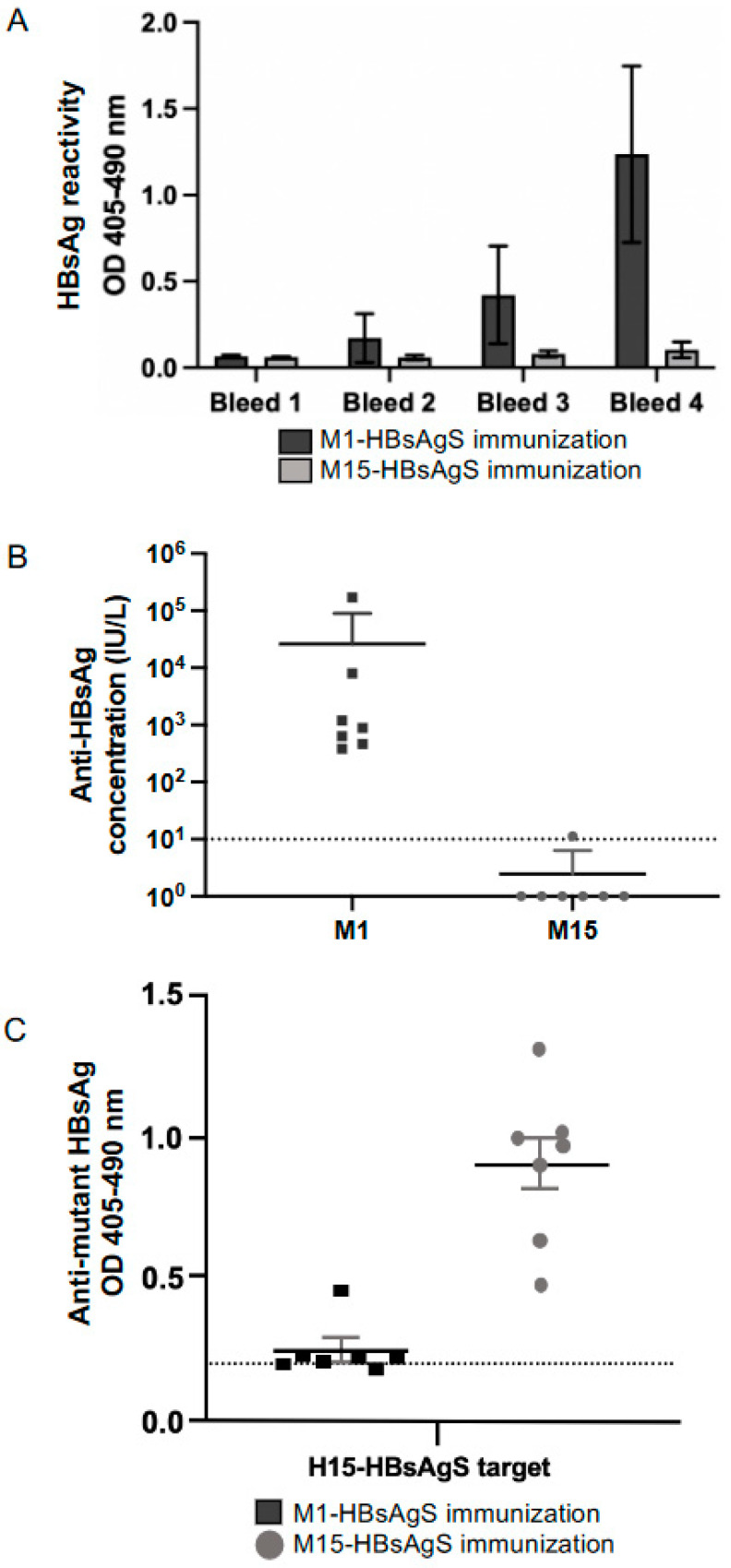
Immunoreactivity of M1- and M15-HBsAgS VLPs. For the generation of antisera, groups of seven BALB/c mice were immunized four times at two-week intervals with 2 μg of M1- and M15-HBsAgS VLPs. The generated antibody responses were assessed using (**A**) an in-house ELISA with yeast-derived wt HBsAgS VLPs as targets, at a 1:250 serum dilution, in duplicate, graphed mean ± standard deviation (SD), (**B**) the anti-HBsAg diagnostic assay Elecsys anti-HBs II (detection threshold <10 IU/L), and (**C**) a mutant HBsAgS-specific ELISA with coated HA-tagged mutant H15-HBsAgS. The cut-off value at 0.2 OD is shown by a dashed line.

To determine whether the mutant M15-HBsAgS VLPs with reduced HBsAgS-specific antigenicity have the capability to induce an anti-HBsAgS-specific immune response, groups of seven BALB/c mice were immunized four times at two-week intervals with 2 μg VLPs in the presence of 2.5 nmol CpG (ODN1668). Every second week, blood samples were collected and tested by ELISA using yeast-derived wt VLPs as targets (Figure 2A). The analysis of the sera taken at the end of the study (day 56) demonstrated that the immunizations with wt M1-HBsAgS VLPs generated detectable anti-HBsAgS antibody titers at a dilution of 1:250, but the M15-HBsAgS VLPs did not. The sera were re-tested at different dilutions using the Elecsys Anti-HBs II assay, allowing the documentation of diagnostic relevant anti-HBsAgS (anti-HBs) antibody titers in international units per liter (Figure 2B). The mice immunized with M1-HBsAgS VLPs developed anti-HBsAgS titers between 171,000 and 381.6 IU/L, in contrast to the mice immunized with M15-HBsAgS VLPs, with anti-HBsAgS antibody levels below detection (<10 IU/L). One mouse developed an anti-HBs titer of 11.3 IU/L (Figure 2B), providing a significant difference regarding HBsAg-specific immunogenicity (*p* = 0.0006). In addition to the loss of HBsAgS-specific antigenicity, the M15-HBsAgS VLPs appear to have lost the ability to induce anti-HBsAgS antibody responses. 

With the absence of significant levels of anti-HBsAgS antibodies in the mice immunized with M15-HBsAgS VLPs, we aimed to confirm that the M15-HBsAgS VLPs had retained their immunogenicity. To prevent reactivity against the myc-tag, we generated HA-tagged versions of the M15-HBsAgS. The H15-HBsAgS VLPs were immobilized by capture on ELISA plates and subsequently analyzed using sera from mice immunized with wt M1-HBsAgS or M15-HBsAgS VLPs. The M15-HBsAgS-immune sera were highly reactive with the H15-HBsAgS VLPs (Figure 2C). In contrast, the sera from mice immunized with the M1-HBsAg VLPs did not show strong antibody responses against mutant HBsAg VLPs (*p* = 0.0002) (Figure 2C). The reactivity of the M15-HBsAgS-specific antisera with the cognate H15-HBsAgS VLP targets indicated that the mutant VLPs had retained immunogenicity. The M15-HBsAgS VLPs represent antigenically distinct but immunogenic HBsAgS scaffolds, which allow comparative immunization studies using M1-HBsAgS versus M15-HBsAgS delivery platforms in the presence of an anti-HBsAgS pre-existing antibody immune response. 

### 3.3. Generation of Chimeric M1- and M15-HBsAgS VLPs with Inserted Foreign NANP9 Antigenic Sequence

**Figure 3:**
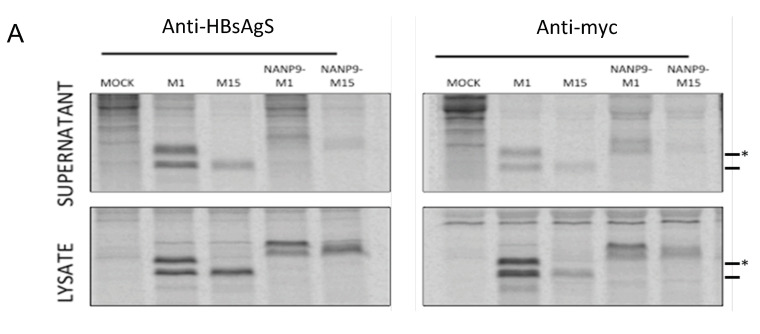

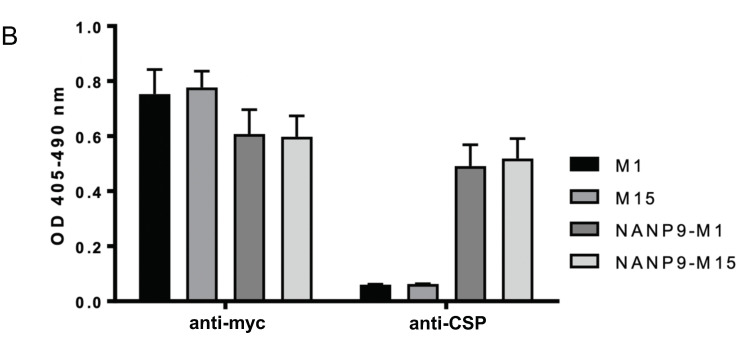
Immuno-detection of NANP9-chimeric HBsAgS proteins by (**A**) immunoprecipitation and (**B**) ELISA. (**A**) ^35^S-radiolabelled M1, M15, NANP9-M1, and NANP9-M15 proteins were immunoprecipitated from the cell culture supernatant (top) and cell lysate (bottom) using anti-HBsAg or anti-myc antibodies before separation via SDS-PAGE. The positions of the M1- and M15-HBsAgS proteins (dash) and M1 glycoprotein (asterisk) are indicated. (**B**) The antigenicity of the M1, M15, NANP9-M1, and NANP9-M15 VLPs was assessed by ELISA; anti-myc antibodies were used to capture VLPs; polyclonal anti-myc or anti-CSP were used to detect VLPs, *n* = 3 in duplicate, graphed mean, and SEM.

To determine the antigenic activity of the wt M1- and mutant M15-HBsAgS VLPs as delivery platforms, nine “NANP” repeats were inserted into the external HBsAgS loop region, as previously described [29]. The NANP9 M1-HBsAgS VLPs have been previously described, and their use in BALB/c mice resulted in the generation of complement-fixing anti-CSP immune sera [29]. The expression of the M1-, NANP9-M1-, M15-, and NANP9-M15-HBsAgS proteins was assessed in HEK293T cells. The ^35^S-labelled proteins were immunoprecipitated with a polyclonal anti-HBsAgS antibody and a monoclonal anti-myc antibody (Figure 3A). Both the M1- and M15-HBsAgS proteins were successfully pulled out of the solution using anti-HBsAgS and anti-myc antibodies, and also immunoprecipitated NANP9-containing HBsAgS proteins of higher molecular weight, as anticipated. The insertion of the NANP9 antigenic sequence into the M1-HBsAgS backbone generated glycosylated and non-glycosylated HBsAgS proteins (28 kDa and 31 kDa), indicating that the insertion of the NANP9 sequence does not compromise glycosylation at the N146 position in the external loop. The NANP9 insertion into the M15-HBsAgS backbone generated non-glycosylated chimeric HBsAgS subunits (28 kDa), due to the absence of the glycosylation site within the M15 mutant sequence. To demonstrate the presence and exposure of the NANP9 insert, anti-myc antibodies were used to capture the chimeric VLPs, which were assayed with anti-CSP antibodies. The NANP9-M1 and NANP9-M15 VLPs were reactive with the anti-CSP rabbit-immune serum, but the parental M1- and M15-HBsAgS VLPs were not (*p* ≤ 0.0001) (Figure 3B). Importantly, the NANP9-M1 and NANP9-M15 were detected to a similar extent (*p* = 0.9129), indicating that the different M1- and M15-HBsAgS contexts have not measurably altered the NANP9 exposure (Figure 3B). The similar antigenic display of NANP9 shows that both VLP candidates can be used as appropriate comparators for subsequent immune sensitization studies. 

### 3.4. Immunogenicity of M1- and M15 HBsAgS Platforms in the Presence and Absence of Pre-Existing Anti-HBsAgS Antibodies

**Figure 4:**
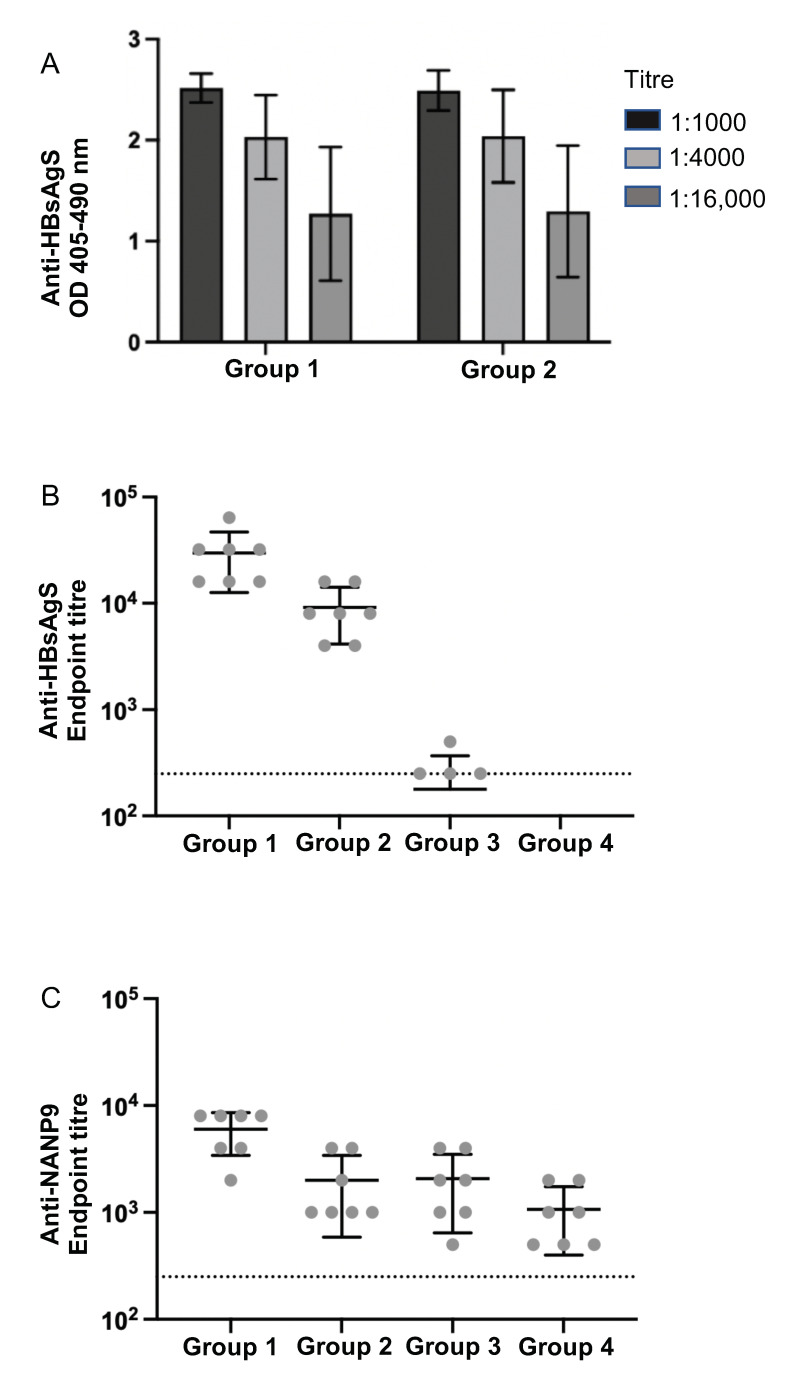
Assessment of immunogenicity of wt and mutant NANP9-VLP platforms in BALB/c mice with a pre-existing anti-HBsAgS antibody response (Groups 1 and 2), or in the absence of a pre-existing anti-HBsAgS (Groups 3 and 4). Test Groups 1 and 3 immunized with NANP9-M1 VLPs with wt HBsAgS backbone, Groups 2 and 4 with NANP9-M15 VLPs with mutant HBsAgS backbone (Supplementary Table S2). (**A**) ELISA to determine anti-HBsAgS antibody titers in Groups 1 and 2 mice before immunization with NANP9-containing VLPs, *n* = 7, in duplicate, graphed mean ± SD. (**B**,**C**) Determination of anti-HBsAgS (**B**) and anti-NANP9 (**C**) antibody endpoint titers. Sera were collected 13 days after the final immunization with chimeric NANP9-containing VLPs and tested for reactivity with (**B**) wt HBsAg VLPs and (**C**) NANP9 peptides, in duplicate, graphed mean ± SD. Titration of individual mouse sera in Supplementary Figure S1.

To determine the NANP9-specific immunogenicity of the NANP9-M1 and NANP9-M15-HBsAgS VLPs in naïve mice compared to mice with a pre-existing anti-HBsAgS immune response, fourteen BALB/c mice were immunized three times with wt HBsAg VLPs produced in *Saccharomyces cerevisiae*. The mice were separated into two groups (Group 1 and Group 2) of matched pairs, so that there was no significant difference in the anti-HBsAgS group titers (*p* ≥ 0.999) (Figure 4A), then used for immunizations with NANP9-M1 (Group 1) and NANP9-M15 (Group 2) HBsAgS VLPs. An additional two groups of mice were used as naïve control groups, in the absence of pre-existing anti-HBsAg antibodies, and used for comparative immunizations with NANP9-M1 VLPs (Group 3) and NANP9-M15 VLPs (Group 4). After the administration of three doses of chimeric vaccines, and 13 days after the last immunization, the final bleed showed that the mice of all the groups developed anti-NANP9 antibodies, with anti-NANP9 peptide titers of at least 1:1000 (Figure 4, Supplementary Figure S1). Interestingly, higher anti-NANP9 antibody titers were generated by the Group 1 animals (pre-existing anti-HBsAgS immunity followed by wt NANP9-M1 VLPs) compared to the Group 2 animals (pre-existing anti-HBsAgS immunity followed by mutant NANP9-M15 VLPs) (Group 1 and Group 2, *p* = 0.0037). The anti-HBsAg titers generated by the Group 1 and Group 2 animals were sustained throughout the experiment, and, by the conclusion of the experiment, the anti-HBsAgS antibody titers were significantly higher in Group 1 compared to Group 2 (*p* = 0.0029), suggesting that the wt M1-backbone provides anti-HBsAgS-specific boosting, while the M15-HBsAgS backbone was less able to do so. Consistently, the NANP9-M1 VLPs achieved higher anti-NANP9 antibody titers in the presence of a cognate pre-existing anti-HBsAgS immune response (Group 1) than in the naïve mice in the absence of a pre-existing anti-HBsAgS response (Group 3) (*p* = 0.0042). Similarly, there were higher anti-HBsAg titers in Group 1 versus Group 3 (*p* = 0.0006). The absence of a detectable level of anti-HBsAg antibodies in the mice immunized with NANP9-M15 VLPs (Group 4), and the lack of a boosting effect in the anti-HBsAg pre-immunized mice (Group 2), suggests the mutations used in the M15 particles reduces HBsAg-specific immunogenicity as well as antigenicity. The data indicate that previous HBsAgS exposure and the presence of an anti-HBsAgS immunity facilitates an enhanced antibody response against foreign epitopes delivered by the cognate HBsAgS VLP platform, and, that the state of prior immune exposure to HBsAgS particles can greatly alter the outcome of subsequent vaccinations based upon similar particle types.

## 4. Discussion

The long-lived and robust anti-HBsAg titers generated by the immunizations with HBsAgS VLPs highlight the suitability of VLPs as vaccines and antigen delivery platforms. The chimeric HBsAgS VLPs have been generated in an attempt to modify this platform for the induction of potent immune responses against foreign inserted sequences. These include chimeric HBsAg vaccines generated against hepatitis C virus (HCV) [26,35], human papillomavirus [36], dengue virus [37], influenza virus [38], human immunodeficiency virus 1 [39,40], and *P. falciparum* [22,41]. The RTS,S/AS01 (Mosquirix) vaccine is currently one of the most advanced chimeric vaccines, with pilot studies in three African countries [23,42] and with a related design R21 also being tested in human trials [43]. The success of the HBV vaccine and its potential to function as an antigen-delivery system has necessitated investigations into the influence of immune sensitization on subsequent antigen exposure. One of the outcomes associated with the success of the HBV vaccine has been its broad implementation in childhood vaccination schedules, resulting in >80% global HBV vaccine coverage [30]. 

To address the impact of the HBsAg platform on immunization outcomes, we mutated the HBsAgS platform to reduce the HBsAgS-specific antigenicity and immunogenicity (Figure 1 and Figure 2) and performed comparative studies using mutant (M15) and wt (M1) HBsAgS backbones. Previous studies have demonstrated that a pre-existing anti-HBsAgS antibody response allows the induction of antibodies specific to the delivered foreign antigenic sequence. VLPs exclusively composed of chimeric HBsAgS proteins with an HCV sequence inserted into the ‘a’-determinant region generated anti-HCV antibody responses in BALB/c mice with a pre-existing anti-HBsAgS immune response [44]. Similarly, VLPs composed of chimeric HBsAg proteins, with the N-terminal transmembrane (tm) region replaced with the tm domain of the HCV envelope 2 proteins and co-assembled with wt HBsAg, allowed successful immunization outcomes in rabbits with pre-existing immunity to HBsAg [45]. Importantly, the outcome of a clinical trial demonstrated that the hybrid RTS,S vaccine achieved higher anti-CSP and anti-HBsAgS antibody responses in children previously vaccinated with the hepatitis B vaccine compared to children in the absence of a hepatitis B vaccination history [46]. To evaluate the impact of the HBsAgS backbone on immunization outcomes in the presence and absence of a pre-existing anti-HBsAgS antibody immune response, mutant HBsAgS platforms were generated with targeted amino acid changes to substantially reduce HBsAgS-specific antigenicity. Only 3/17 monoclonal antibodies retained the ability to bind to their cognate epitopes (Figure 1C). Consistently, with the loss of HBsAgS-specific antigenicity, the M15-HBsAgS VLPs are compromised in inducing an anti-HBsAgS antibody response (Figure 2B) but retained immunogenicity and induced antibody responses specific for the mutant HBsAg subunits. The antisera generated by M15-VLP immunizations are reactive with cognate H15-VLPs in contrast to antisera generated with wt M1-VLPs (Figure 2C). Importantly, both the wt M1-HBsAgS and mutant M15-HBsAgS VLPs supported NANP9 exposure and allowed comparative immunization studies. The detection of M1- and M-15 HBsAgS-NANP9 particles by an anti-CSP immune serum suggests that the NANP9 presentation within the external loop retained its exposure in the mutant HBsAgS proteins (Figure 3B). 

The NANP9-M1 and NANP9-M15 VLPs appear to show similar abilities to display the inserted NANP9 sequence; however, they differ in their ability to generate anti-NANP9 responses in the context of previous HBsAgS exposure. The presence of anti-HBsAgS antibodies recognizing their cognate wt platform has a positive impact on subsequent vaccine responses. The improved B cell response due to carrier priming is possibly facilitated by the HBsAgS-specific antigenic density provided by the chimeric HBsAgS VLPs. Jegerlehner et al. [13] showed that carrier-induced epitopic suppression could be overcome by a higher epitope density [13]. Using VLPs as immunological carriers for chemically coupled peptide epitopes demonstrated that anti-VLP-specific pre-existing immune responses resulted in decreased peptide-specific antibody titers. The suppression of the anti-peptide immune response could be overcome by high coupling densities of the test peptide, higher doses of the conjugate vaccine, or repeated vaccinations [13,14]. Related studies investigating anti-polysaccharide antibody responses demonstrated that immunization with the carrier molecule enhances the response to conjugate vaccines, probably by increasing the number of carrier-specific T cells, which provide help for the expansion of polysaccharide-specific B-lymphocytes. Contrarily, a low ratio of hapten to the carrier may favor suppression [9]. Secretion-competent HBsAgS proteins, including secretion-competent chimeric HBsAgS proteins, expressed in higher eukaryotic cells assembled into compact VLPs [25,29], and yeast-derived HBsAgS assembly into highly dense particles, probably during the downstream processing [47], providing a high antigenic density and hence, possibly facilitate carrier priming. The high density of the HBsAgS subunits may have promoted the immune response against the inserted foreign epitopes, even for the RTS,S vaccine, which provides a ratio CSP-polytope-HBsAg fusion protein to wt HBsAgS protein 1:4 [22,46]. Further improvements of the vaccine are possibly achievable by biochemical modifications, such as hyperglycosylation, to improve the interaction with immune-competent cells [31,48,49]. Vaccine strategies based on modifiable scaffolds with broad global vaccine coverage, such as the protective HBsAgS vaccine, may provide an advantage to generate strong immune responses against selected targets with unmet medical needs. 

## Data Availability

Data sharing not applicable. No new data were created or analyzed in this study. Data sharing is not applicable to this article.

## Supplementary Materials

The following supporting information can be downloaded at: https://www.mdpi.com/article/10.3390/v15020313/s1, Supplementary Figure S1: Anti-HBsAgS and anti-NANP9 activity of individual mouse sera. Supplementary Table S1: Differences between the wild type (wt) HBsAg protein and the mutant (M15) protein. Supplementary Table S2: Immunization schedule for mouse groups with or without induced pre-existing anti-HBsAgS.
