# Supplementary material for: Immunogenicity of Wild Type and Mutant Hepatitis B Surface Antigen Virus-like Particles (VLPs) in Mice with Pre-Existing Immunity against the Wild Type Vector
Source: Viruses. 2023 Jan 23;15(2):313. doi: 10.3390/v15020313 (PMC9963944; doi:10.3390/v15020313)
Supplement: Supplementary file 1 [file viruses-15-00313-s001.zip › viruses-2167819-supplementary.pdf]

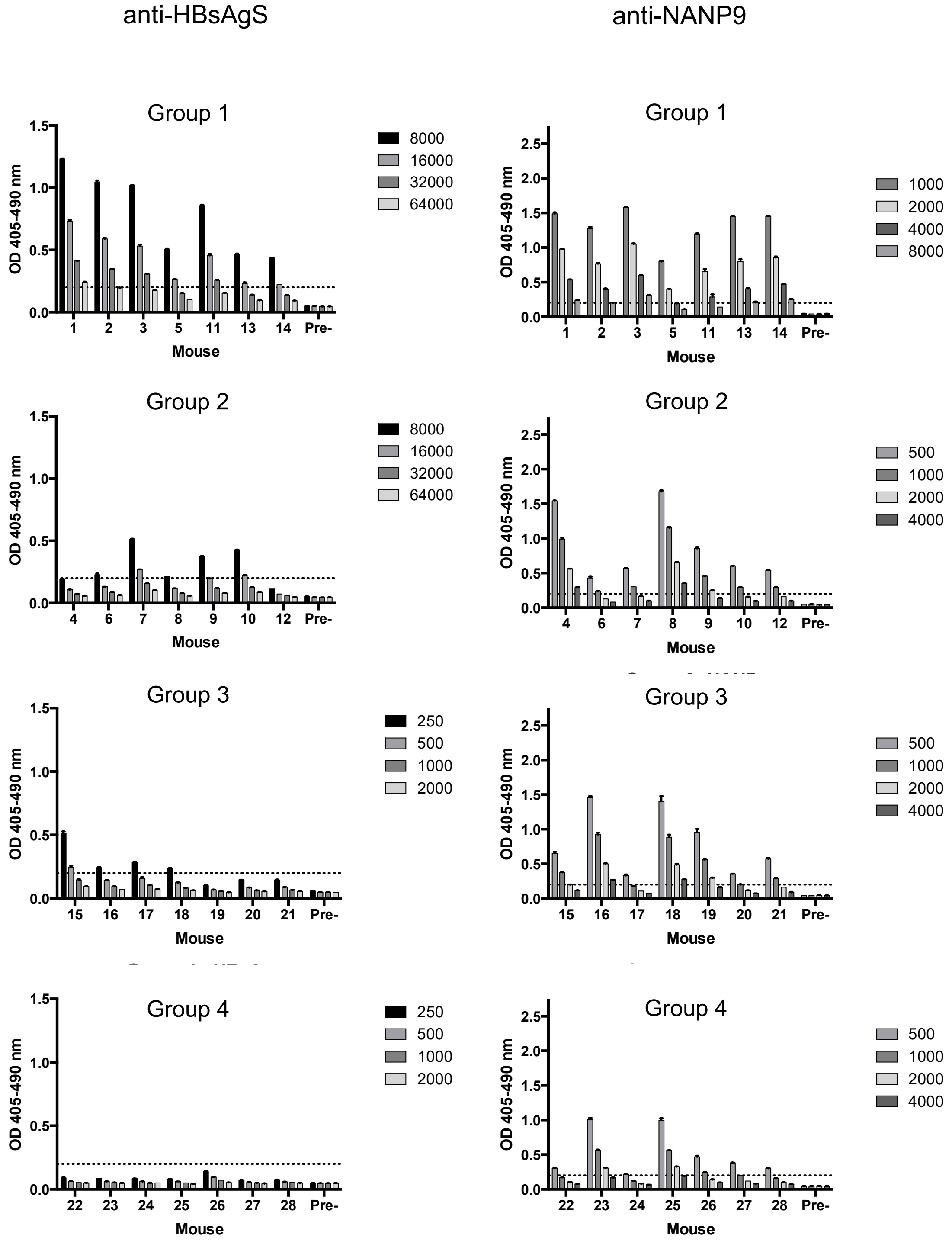


Supplementary Figure 1

Anti-HBsAgS and anti-NANP9 activity of individual mouse sera from mice with (group 1 and 2) or without (group 3 and 4) pre-existing anti-HBsAgS immunity. The number of the individual mice are shown at the x-axis, the OD values of the ELISA against wt VLPs or NANP9 peptides are given at the y-axis. “Pre-” stands for pre-immune serum. Dotted line indicates OD of 0.2, ODs >0.2 are considered positive. Note: for clarity different dilution series are used to allow display of the different dynamic range of anti-HBsAg and anti-NANP9 responses across different groups. Graphed mean and SEM in duplicate.

Supplementary Table 1

Differences between the wild type (wt) HBsAg protein and the mutant (M15) protein. “x” represents deletion. The positions refer to the wt HBsAgS protein (genotype D, serotype *ayw*) with 226 amino acids.

| Name | Amino acid positions | | | | | | | |
|---|---|---|---|---|---|---|---|---|
| M1 (wt) | **P120** | **C121** | **R122** | **C124** | **D144** | **G145** | **N146** | **C147** |
| M15 | T | A | G | A | E | R | x | A |

Supplementary Table 2

Immunization schedule for groups with induced pre-existing anti-HBsAgS immune response (group 1 and 2) and in the absence of a pre-existing anti-HBsAgS immune response (group 3 and 4). Wild type (wt) VLP stands for yeast-derived virus-like particles (VLPs) (serotype *ayw*). M1 and M15 represents myc-tagged wt and mutant HBsAgS VLPs, respectively. NANP9 indicates the insertion of the nine-mer NANP repeat into the HBsAgS external loop region.

| | **Pre-immunity** | | | **Trial** | | |
|---|---|---|---|---|---|---|
| | **Day 0** | **Day 14** | **Day 28** | **Day 42** | **Day 56** | **Day 70** |
| **Group 1** | WT VLP | WT VLP | WT VLP | NANP9-M1 | NANP9-M1 | NANP9-M1 |
| **Group 2** | WT VLP | WT VLP | WT VLP | NANP9-M15 | NANP9-M15 | NANP9-M15 |
| **Group 3** | - | - | - | NANP9-M1 | NANP9-M1 | NANP9-M1 |
| **Group 4** | - | - | - | NANP9-M15 | NANP9-M15 | NANP9-M15 |
